# Vonoprazan versus lansoprazole in the treatment of artificial gastric ulcers after endoscopic submucossal dissection: a randomized, open-label trial

**DOI:** 10.1186/s12876-021-01822-5

**Published:** 2021-05-22

**Authors:** Daisuke Kawai, Ryuta Takenaka, Mikako Ishiguro, Shotaro Okanoue, Tatsuhiro Gotoda, Yoshiyasu Kono, Koji Takemoto, Hirofumi Tsugeno, Shigeatsu Fujiki

**Affiliations:** Department of Gastroenterology, Tsuyama Chuo Hospital, 1756, Kawasaki, Tsuyama, Okayama 708-0841 Japan

**Keywords:** Vonoprazan, Lansoprazole, Postoperative bleeding, Endoscopic submucosal dissection, Gastric neoplasm

## Abstract

**Background:**

Vonoprazan is more potent and longer acting than traditional proton pump inhibitor. Although vonoprazan is expected to be superior to proton pump inhibitor, its efficacy in the treatment of gastric ulcers following endoscopic submucosal dissection (ESD) is not fully understood. The aim of this study was to evaluate the effectiveness of vonoprazan in artificial ulcer healing following ESD.

**Methods:**

Patients with gastric tumors were randomly assigned to the vonoprazan group (group V) or lansoprazole group (group L) after ESD. Patients received intravenous lansoprazole (30 mg) twice on the day of ESD. Thereafter, patients were treated with vonoprazan (20 mg/day) in group V or lansoprazole (30 mg/day) in group L. Esophagogastroduodenoscopy was performed 4 and 8 weeks after the ESD.

**Results:**

A total of 168 patients were analyzed. The 4-week healing rate for artificial ulcer was not significantly higher in group V versus group L (17/85, 20.0% vs. 14/83, 16.9%, respectively). In addition, there were no significant differences between the 4-week shrinkage rates between the two groups. Postoperative bleeding occurred in none of the patients in group V and three in group L. One patient in group V presented delayed perforation 2 days after ESD.

**Conclusions:**

Vonoprazan might not be superior to lansoprazole in the healing of artificial gastric ulcer after ESD.

*Trial registration*: University hospital Medical Information Network (registration number: UMIN000016642), Registered 27 February 2015, https://www.umin.ac.jp/ctr/index-j.htm.

## Background

Endoscopic submucosal dissection (ESD) for early gastric cancer has been widely accepted and is a well-established procedure in Eastern and Western countries [1–4]. ESD provides higher rate of *en bloc* and R0 resection rate, but is occasionally associated with some complications, such as bleeding and perforation [5]. Conventional proton pump inhibitors (PPIs) have been widely used for treating ESD-induced gastric ulcers. Although PPIs support to heal such lesions, some ulcers fail to heal; therefore, a more effective therapy is warranted.

Vonoprazan is a novel suppressant of gastric acid secretion and an active potassium-competitive acid blocker (P-CAB) [6]. Similar to PPIs, P-CABs inhibit gastric H+/K+-ATPase. Unlike PPIs, P-CABs inhibit the enzyme in a K+-competitive and reversible manner. The inhibitory effect of vonoprazan on gastric acid secretion is largely unaffected by ambient pH. Therefore, vonoprazan is more potent with a more long-lasting effect than that of PPIs [7, 8]. Vonoprazan is expected to be superior to PPIs, and lead to earlier healing of ESD-induced gastric ulcers versus conventional PPI-based therapy. However, its efficacy in treating these ulcers remains unclear. Hence, in this study, we aimed to evaluate the effectiveness of vonoprazan in healing artificial ulcers after ESD.

## Methods

This study was a prospective, single-center, randomized, open-label controlled trial (RCT). The study protocol was approved by the local ethics committee of Tsuyama Chuo Hospital, Tsuyama, Japan and registered with the University hospital Medical Information Network (URL: https://www.umin.ac.jp/ctr/index-j.htm; registration number: UMIN000016642). Written informed consent was provided by each patient.

Patients with gastric tumors were enrolled between April 2015 and December 2017. Inclusion criteria for ESD were as follows: differentiated mucosal cancers without ulceration regardless of the lesion’s size; differentiated mucosal cancers ≤ 30 mm with ulcer findings; mucosal cancers with undifferentiated histology ≤ 20 mm without ulceration; adenoma suspicious for mucosal cancers; and neuroendocrine tumors grade 1 ≤ 10 mm confined to submucosal layer. The exclusion criteria were: remnant stomach, administration of antithrombotic agents, non-steroidal anti-inflammatory drugs (NSAIDs) and steroids; occurrence of complication during ESD; allergy to lansoprazole or vonoprazan; and unwillingness to participate in the study. Following ESD, patients were randomly assigned to the vonoprazan group (group V) or the lansoprazole group (group L) through the minimization method using Kullback–Leibler divergence [9]. The ESD ulcer index was used to balance continuous variables. On the day of ESD, patients received 30 mg of lansoprazole twice intravenously. From postoperative day 2, patients in groups V and L received 20 mg/day of vonoprazan and 30 mg/day of lansoprazole for 8 weeks, respectively.

**Fig. 1:**
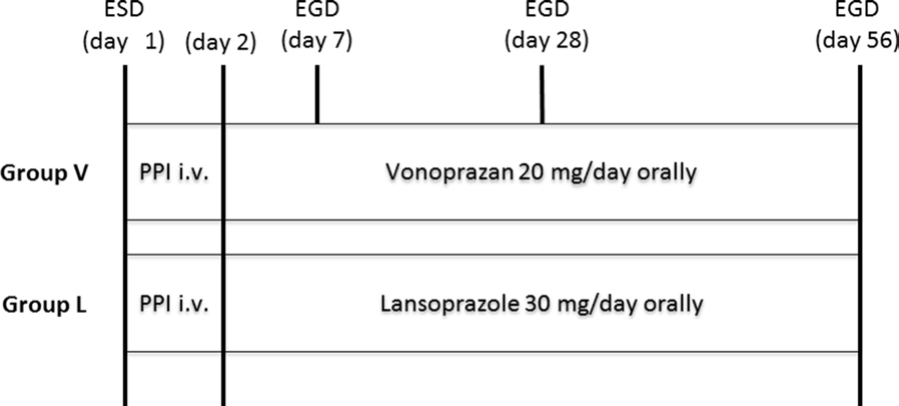
Study protocol. ESD, endoscopic submucosal dissection; EGD, esophagogastroduodenoscopy; group V, vonoprazan group; group L, lansoprazole; PPI, proton pump inhibitor; i.v., intravenous injection Flow chart of patients. ESD, endoscopic submucosal dissection

**Fig. 2:**
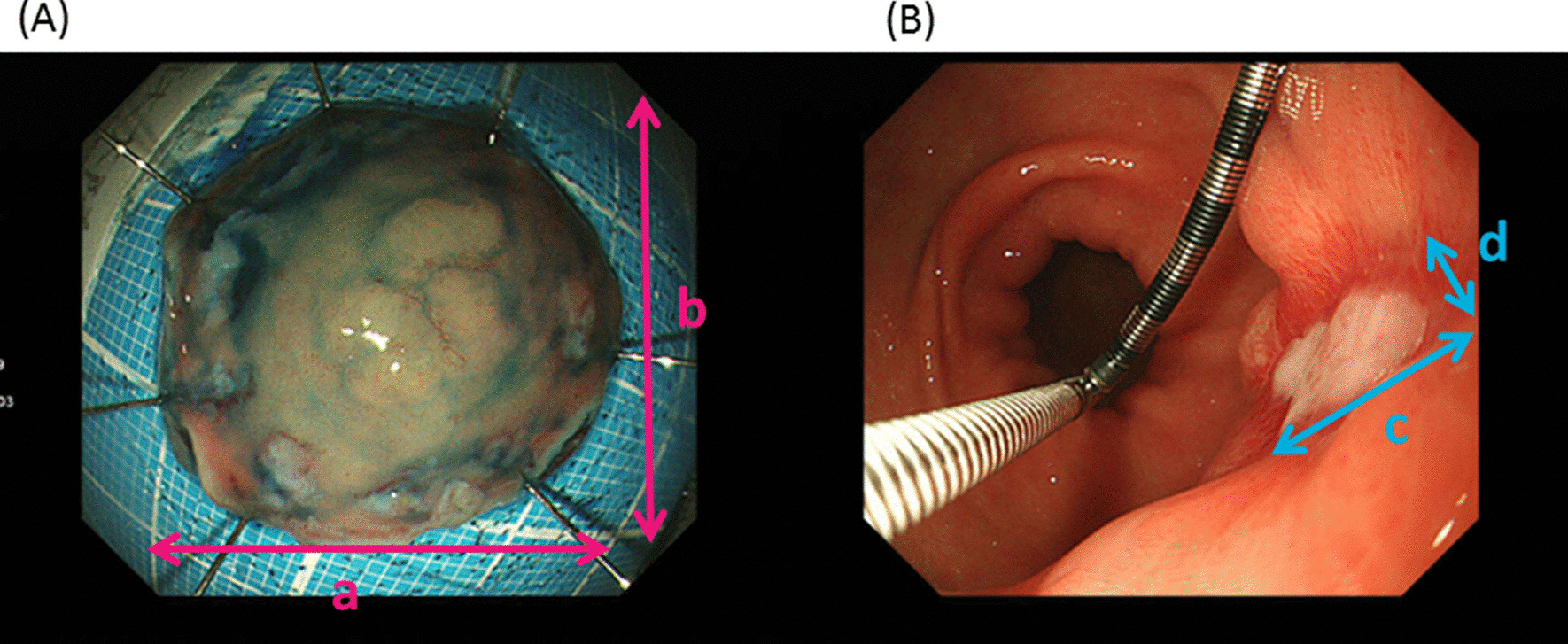
**A** The ESD ulcer index is calculated by multiplying the length (a) by the width (b) of the resected specimen. **B** The 4- and 8-week ulcer indices were calculated by multiplying the length (c) by the width (d) of the artificial ulcer at 4 and 8 weeks, respectively

Esophagogastroduodenoscopy was performed 4 and 8 weeks after the ESD (Fig. 1). During the follow-up endoscopy, the artificial ulcer was evaluated using a gastric ulcer stage system [10], and the length and width of the artificial ulcer were evaluated with measure forceps (M2-2C, M2-3U or M2-4K, Olympus Co, Japan). The ulcer healing was defined as scarring at S1 or S2. The ESD ulcer index was calculated by multiplying the length by the width of the resected specimen (Fig. 2A). The 4- and 8-week ulcer indices were also calculated by multiplying the length by the width of the artificial ulcer at 4 and 8 weeks after ESD, respectively (Fig. 2B). The shrinking rate was defined as [1-(the ulcer index)/(the ESD ulcer index)] × 100 (%).

The primary endpoint was the healing rate of the artificial ulcer at 4 weeks after ESD. The secondary endpoints were: the healing rate at 8 weeks; shrinkage rates of the artificial ulcers at 4 and 8 weeks; and complications, such as post-operative bleeding and delayed perforation.

The healing rate of ulcers after the 4-week administration of PPIs was reported as 11–35% [11, 12]. According to our previous data, the healing rate after a 4-week administration of PPIs was 24%. A 20% improvement observed following the administration of vonoprazan was considered clinically effective. It was estimated that 82 cases were required in each group to have a power of 80% for detection of a difference at an α = 0.05 level of significance using Fisher’s exact test. Assuming a study drop-out rate of approximately 10%, 90 cases were enrolled in each group.

Differences between the two groups were determined using the chi-squared test or Fisher’s exact test for discontinuous variables and the Mann–Whitney U test for continuous variables. Statistical analyses were performed with the JMP (version 13) software package (SAS Institute, Cary, NC, USA). *P*-values < 0.05 denoted statistically significant differences between groups.

## Results

**Fig. 3:**
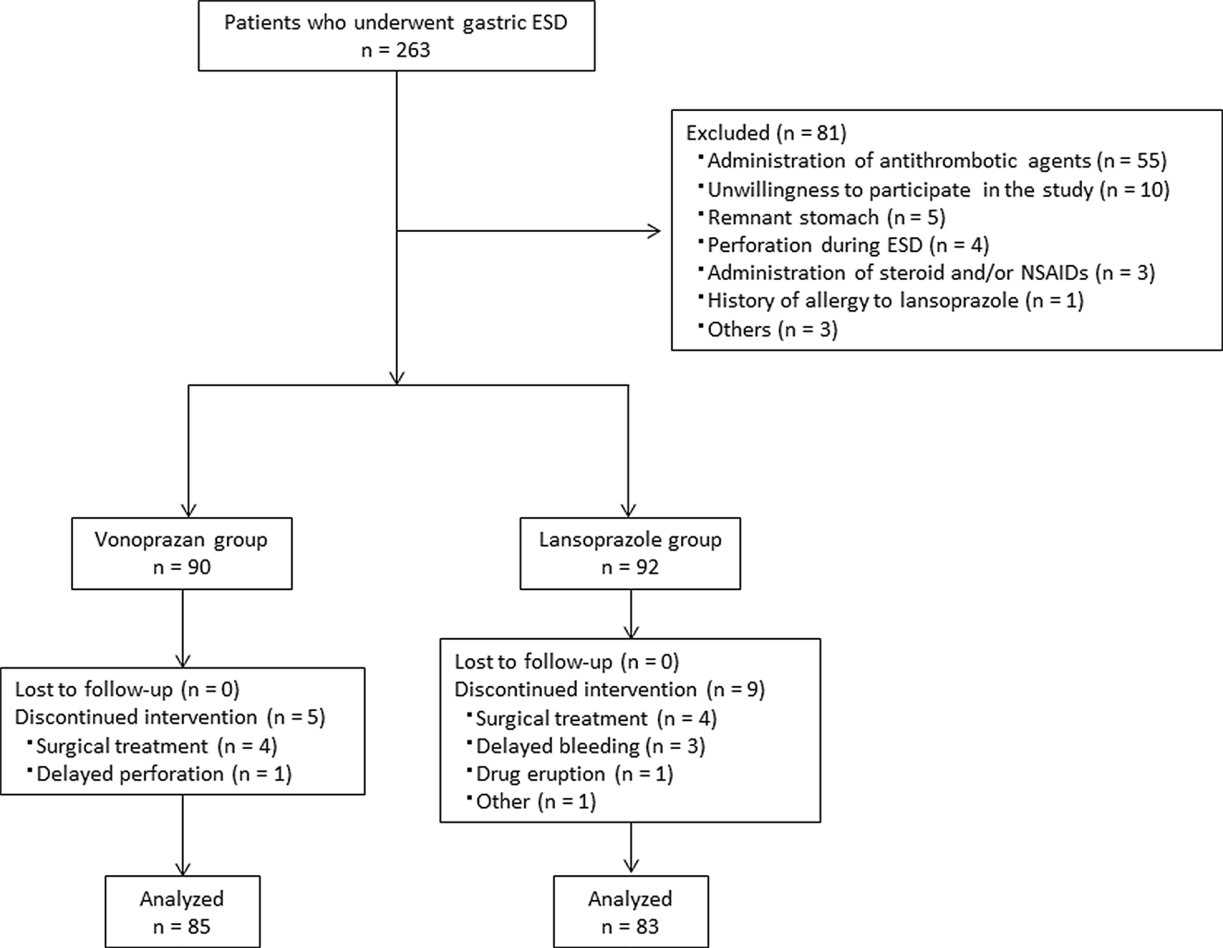
Flow chart of patients. ESD, endoscopic submucosal dissection

Of the 263 patients who underwent gastric ESD from April 2015 to December 2017, 182 patients (90 in group V and 92 in group L) were eligible to participate in the study. Fourteen patients were excluded during follow-up mainly because of complications or the need for additional surgery. Finally, 85 and 83 patients were allocated to group V and group L, respectively (Fig. 3). The age, gender, status of *Helicobacter pylori* infection, tumor location, and ESD ulcer index of the two groups did not differ significantly (Table 1). The 4-week healing rate of artificial ulcers was not significantly higher in group V versus group L (17/85, 20.0% vs. 14/83, 16.9%, respectively). Furthermore, there was no significant difference between the 4-week shrinkage rates noted in groups V and L. Postoperative bleeding was observed in three patients of group L and none of the patients in group V. One patient in group V presented delayed perforation 2 days after ESD (Table 2).

**Table 1:** Clinical characteristics of the study participants

	Vonoprazan group n = 85	Lansoprazole group n = 83	*P* value
Age (years), median (range)	73 (47–89)	73 (33–90)	0.96
Sex, male, n (%)	63 (74)	58 (70)	0.54
*Habits*			
Smoking, n (%)	16 (19)	16 (19)	0.94
Alcohol, n (%)	36 (42)	34 (41)	0.86
*Anti-Helicobacter pylori IgG antibody*			
Positive, n (%)	30 (35)	31 (37)	0.78
*PPI use prior to treatment			
Yes, n (%)	24 (28)	18 (22)	0.33
*Comorbidities*			
Hypertension, n (%)	56 (66)	44 (53)	0.089
Diabetes mellitus, n (%)	18 (21)	14 (17)	0.48
Ischemic heart disease, n (%)	0 (0)	2 (2)	0.24
Cerebral infarction, n (%)	0 (0)	1 (1)	0.49
*Location of lesion*			
Upper third, n (%)	9 (11)	11 (13)	0.28
Middle third, n (%)	35 (41)	42 (51)	
Lower third, n (%)	41 (48)	30 (36)	
Lesion size (mm), median (range)	10 (2–54)	10 (2–40)	0.85
*Preoperative histological diagnosis*			
Adenoma, n (%)	19 (22)	27 (32)	0.20
Differentiated adenocarcinoma, n (%)	64 (75)	52 (63)	
Undifferentiated adenocarcinoma, n (%)	2 (2)	2 (2)	
Neuroendocrine tumor grade 1, n (%)	0 (0)	2 (2)	
Ulcer scar in tumor, n (%)	6 (7)	7 (8)	0.74
^†^ESD ulcer index (mm^2^), median (range)	875 (260–4000)	875 (238–4180)	0.59

*PPI, proton pump inhibitor; ^†^ESD, endoscopic submucosal dissection

**Table 2:** Clinical outcomes of the endoscopic submucosal dissection induced ulcers

	Vonoprazan group n = 85	Lansoprazole group n = 83	*P*-value
Healing of the artificial ulcer at 4 weeks, n (%)	17 (20.0)	14 (16.9)	0.60
Healing of the artificial ulcer at 8 weeks, n (%)	66 (77.6)	70 (84.3)	0.27
Median shrinkage rate of the artificial ulcer at 4 weeks, % (range)	96.3 (18.2–100)	95.1 (39.3–100)	0.68
Median shrinkage rate of the artificial ulcer at 8 weeks, % (range)	100 (92.7–100)	100 (89.3–100)	0.35
*Complications*			
Delayed perforation, n (%)	1 (1)	0 (0)	1.00
Post-operative bleeding, n (%)	0 (0)	3 (4)	0.12

## Discussion

In this RCT, we attempted to prove the superiority of vonoprazan to lansoprazole in the healing rate after gastric ESD in patients stratified according to the ESD ulcer index. Unfortunately, vonoprazan was not superior to lansoprazole in terms of the healing rates and shrinkage rates of artificial gastric ulcers at 4 and 8 weeks after ESD. Furthermore, there were no significant differences in the occurrence of postoperative bleeding and delayed perforation between the two groups.

Some RCTs suggested that vonoprazan was as effective as PPIs in the treatment of ESD-induced ulcer [13–15]. Our results were consistent with those previously reported, in which the healing rates at 4 weeks in the vonoprazan groups ranged 7.4–20.9%. Our hypothesis was that the strong and rapid inhibition of gastric acid secretion by vonoprazan may enhance the healing of artificial ulcers. However, the effect of ulcer shrinkage in the vonoprazan group was similar to that noted in the PPI group. There may be three reasons for this result. Firstly, acid suppression by both vonoprazan and lansoprazole are excellent at shrinking artificial ulcers. Secondly, other factors than acid suppression are involved in the rapid resolution of ulcer. These factors include the existence of ulcer scar, ulcer area, ulcer site, blood coagulation status, *Helicobacter pylori* infection, and other comorbidities. Thirdly, the patients taking antithrombotic agents, NSAIDs and steroids leading to mucosal injury were excluded in this trial.

On the other hand, some studies concluded that vonoprazan was superior to PPIs for healing ESD-induced ulcers [16–19]. However, there were few prospective, randomized controlled studies conducted. Tsuchiya et al. reported that the vonoprazan group had a significantly superior shrinkage rate at 8 weeks; however, there was no significant difference observed in the rate of postoperative bleeding [16]. In this prospective study, the shrinkage rates until 6 weeks were not significantly different. However, the 8-week shrinkage rate was significantly higher in the vonoprazan group versus the PPI group. In a systematic review and network meta-analysis, the effect of vonoprazan at 8 weeks was superior to that of PPIs for the treatment of artificial ulcers following ESD [20]. However, another meta-analysis reported that the healing rate at 8 weeks was significantly higher in the PPI group versus the vonoprazan group [21]. Thus, the effect of vonoprazan remains controversial and further RCTs are warranted. Although the primary endpoint did not meet, we believe the findings of our study are meaningful.

In this investigation, only three patients in the PPI group developed postoperative bleeding. In the 5 RCTs (including this trial) conducted thus far, the rate of postoperative bleeding in the vonoprazan group range 0–5.4%. Although the backgrounds of the study differ, the postoperative bleeding rate was equal or lower in the vonoprazan group versus the PPI group in all studies. Hamada et al. showed that vonoprazan efficaciously reduced the delayed bleeding rate in patients with an ESD-induced gastric ulcer in comparison with the threshold rate recorded using binomial testing [22]. A larger-scale study with postoperative bleeding as its primary endpoint or meta-analysis using RCTs may prove the efficacy of vonoprazan against this complication.

There were some limitations in this study. Firstly, this study was conducted in a single center in Japan and sample size was small. Secondly, patients receiving antithrombotic agents were excluded. As postoperative bleeding was associated with administration of antithrombotic agents, its rate may be underestimated in this study.

## Conclusions

Vonoprazan might not be superior to lansoprazole in the healing of artificial gastric ulcers after ESD in patients without taking antithrombotic agents, NSAIDs and steroids. However, our data are meaningful because the effect of vonoprazan remains controversial and larger-scale RCTs are required to verify the present findings.

## Data Availability

All data generated or analyzed during this study are available from the corresponding author on reasonable request.

## Abbreviations

ESD: Endoscopic submucosal dissection
NSAIDs: Non-steroidal anti-inflammatory drugs
P-CAB: Potassium-competitive acid blocker
PPI: Proton pump inhibitor
RCT: Randomized controlled trial
